# A Fast, Convenient, Polarizable Electrostatic Model for Molecular Dynamics

**DOI:** 10.1021/acs.jctc.3c01171

**Published:** 2024-01-19

**Authors:** Liangyue Wang, Michael Schauperl, David L. Mobley, Christopher Bayly, Michael K. Gilson

**Affiliations:** † Department of Chemistry and Biochemistry, University of California, San Diego, California 92093, United States; ‡ HotSpot Therapeutics, Inc., Boston, Massachusetts 02210, United States; § Department of Pharmaceutical Sciences, University of California, Irvine, California 92697, United States; ∥ OpenEye Scientific, Cadence Molecular Sciences, Santa Fe, New Mexico 87508, United States; ⊥ Skaggs School of Pharmacy and Pharmaceutical Sciences, University of California, San Diego, California 92093, United States

## Abstract

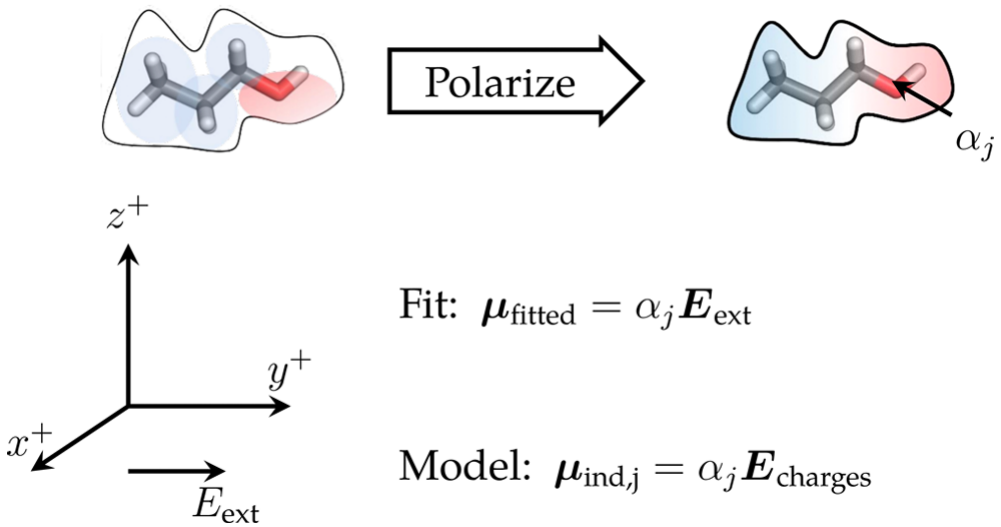

We present an efficient polarizable electrostatic model, utilizing typed, atom-centered polarizabilities and the fast direct approximation, designed for efficient use in molecular dynamics (MD) simulations. The model provides two convenient approaches for assigning partial charges in the context of atomic polarizabilities. One is a generalization of RESP, called RESP-dPol, and the other, AM1-BCC-dPol, is an adaptation of the widely used AM1-BCC method. Both are designed to accurately replicate gas-phase quantum mechanical electrostatic potentials. Benchmarks of this polarizable electrostatic model against gas-phase dipole moments, molecular polarizabilities, bulk liquid densities, and static dielectric constants of organic liquids show good agreement with the reference values. Of note, the model yields markedly more accurate dielectric constants of organic liquids, relative to a matched nonpolarizable force field. MD simulations with this method, which is currently parametrized for molecules containing elements C, N, O, and H, run only about 3.6-fold slower than fixed charge force fields, while simulations with the self-consistent mutual polarization average 4.5-fold slower. Our results suggest that RESP-dPol and AM1-BCC-dPol afford improved accuracy relative to fixed charge force fields and are good starting points for developing general, affordable, and transferable polarizable force fields. The software implementing these approaches has been designed to utilize the force field fitting frameworks developed and maintained by the Open Force Field Initiative, setting the stage for further exploration of this approach to polarizable force field development.

## 1 Introduction

Molecular dynamics (MD) simulations can provide qualitative and quantitative insights into molecular systems without the expense of laboratory experiments. They are widely used to elucidate biomolecular mechanisms and speed up the discovery of new medications. An MD simulation requires a mathematical function called a force field to estimate the forces among atoms of a system, and these forces are used to predict the trajectory of the atoms over time. The design of a force field involves trade-offs between accuracy, essential to generate a conformational ensemble similar to the real-world one, and computational speed, needed for efficient sampling of thermodynamically and kinetically relevant configurations of the molecular system. In order to achieve a suitable speed-accuracy balance, a typical force field uses a theoretically motivated but ultimately empirical functional form with an array of adjustable parameters that are tuned against reference data.

The force fields most widely used today approximate the electrostatic interactions of a molecular system in terms of Coulombic interactions among fixed, atom-centered, partial charges.^1−4^ The partial charges of a given molecule are typically chosen to replicate the electrostatic potential (ESP) generated by the molecule in a vacuum, as computed by reference quantum mechanical (QM) methods.^1,5−7^ Such so-called fixed charge electrostatic models are computationally efficient and have been remarkably useful. However, their accuracy is limited by their neglect of the field-dependent electronic polarization, i.e., changes in the molecule’s electron distribution in space in response to changes in an electric field. Including an explicit treatment of electronic polarizability is expected to improve accuracy, especially in molecular systems with large fields due to ions^8−15^ and in settings where polar molecules move between media of high dielectric constant (e.g., water) and low dielectric constant (e.g., a cell membrane), because changes in dielectric constant lead to changes in the self-polarization of polar molecules.^15−17^

Indeed, the electric field at an atom changes constantly during a simulation because of the movements of other atoms with their partial charges, and these changes induce continual changes in the charge distribution within a molecule and hence its interatomic forces. These fast charge rearrangements are responsible for the fact that nonpolar organic liquids have static dielectric constants of about 2 and that all organic liquids have high-frequency dielectric constants of about 2.^18−20^ Polar liquids have higher static dielectric constants because they have permanent dipole moments, which confer orientational polarizability, but this responds more slowly than electronic polarization, so the dielectric constant of a polar liquid falls off with increasing frequency of a time-varying external electric field.^18−20^ Electronic polarization also accounts for the fact that polar molecules become less polar when moved from a high-dielectric constant medium to a low-dielectric medium (or vacuum), where the reaction field in the molecule is weaker.^21^ A fixed charge force field cannot account for this change and is thus expected to lose accuracy when used to model processes where the dielectric environment of a molecule changes much. This may also help explain why water molecules treated as polarizable have a greater tendency to occupy nonpolar binding sites than water molecules treated with fixed charges.^22^

We therefore expect that integrating electronic polarizability into a well-parametrized force field can lead to improved accuracy.^16^ However, the success of such an advance hinges not only on accuracy but also on how easily the force field can be applied to new molecules and on how well it maintains the efficiency of MD simulations. Pioneering work along these lines dates back to at least the 1970s,^23^ and most current polarizable force fields are based on one of three models: induced dipoles, Drude oscillators, or fluctuating charges (FQs).

The induced dipole model, which is used in the present study, places a linear, point-polarizability at the center of each atom.^23,24^ The field at atom *j*, ***E***_*j*_ leads to an induced dipole as
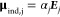
1where α_*j*_ represents either a scalar polarizability or a polarizability tensor, and in general ***E***_*j*_ = ***E***_*j*,ext_ + ***E***_*j*,perm_ + ***E***_*j*,ind_, where the three contributions are the external field (if present), the field due to permanent partial atomic charges on other atoms, and the field due to induced dipoles on other atoms. Because the value of each atom’s induced dipole depends on the induced dipoles present on other atoms, induced dipoles are usually solved with a self-consistent field (SCF) method, also termed a mutual polarization method. This fully accounts for the contribution of the induced electric field (***E***_*j*,ind_) to the induced dipoles on other atoms. The well-developed AMOEBA force field^25^ uses this approach, along with a more detailed multipole representation of the permanent charge distribution. The PHAHST polarizable potentials, designed for material simulations, also utilize the induced dipole model.^26^ The complexity of solving for induced dipoles by SCF methods may be reduced by an empirical extrapolation scheme developed by Simmonett et al.^27^ An even simpler approach is afforded by the direct approximation of electronic polarization,^28^ where atomic polarizabilities do not “feel” the fields generated by other induced dipoles but only the fields generated by partial charges and the external field (if any); the approximation is thus ***E***_*j*_ ≈ ***E***_*j*,ext_ + ***E***_*j*,perm_. This approach was utilized in the iAMOEBA polarizable water model,^29^ which successfully replicates many physical properties of water. However, the direct approximation has not yet been applied to a wider range of systems. In this work, we extended the usage of the fast direct approximation in various small organic molecules by deriving appropriate force field parameters in the context of direct polarization.

The Drude polarizable force field^30^ is the polarizable version of the CHARMM family of force fields.^2,31,32^ It accounts for polarizability by attaching an auxiliary charged particle to each polarizable atomic center with a harmonic spring.^33^ A small charge and mass are transferred from the parent atoms to the Drude particles, allowing them to be treated as dynamic particles that move around their parent atoms. A key advantage of this approach is that the movements of the Drude particles can be accommodated in the usual MD scheme with little modification, rather than requiring the added code needed to handle the inducible dipole model above. However, the added particles make for a somewhat more complex system; a dual-thermostat extended Lagrangian algorithm is used to keep the Drude particles at a very low temperature (∼1 K), and a 1 fs time step is employed because the Drude oscillators have high natural frequencies. Following the introduction of the Drude polarizable water model in 2003,^33^ the Drude polarizable force field has successfully been extended to proteins, deoxyribonucleic acid, lipids, and carbohydrates.^30,34,35^

The FQ model describes polarization by allowing charge to flow between atoms in response to the electric field,^36−38^ so neither inducible dipoles nor added charge centers are required. The modified charges are typically obtained via an electronegativity equalization approach,^39,40^ whose solution can be somewhat time-consuming. Because charges can flow only along the direction of bonds, the FQ model does not describe out-of-plane polarization. Nonetheless, the FQ representation can afford accuracy competitive with the induced polarizable representation.^41^

The present study builds on prior advances to propose two polarizable electrostatic models intended to afford a favorable balance of accuracy, ease of use, and computational speed. Both models assign each atom a typed, atom-centered, linear, isotropic polarizability. These empirical atomic polarizabilities are derived from ESP responses when an imposed external electric field is present. This method has been widely employed in different flavors of the induced dipole model, some examples are included in refs (42–46). In one model, partial charges are assigned with RESP^5^-type fitting to QM ESPs in the context of the typed polarizabilities. In the second model, the AM1-BCC^6,7^ method is used, but with a new set of BCCs trained to replicate QM ESPs in the context of our typed polarizabilities. These assignment methods allow the facile assignment of polarizabilities and compatible partial charges to a wide range of compounds. In addition, we make consistent use of the direct polarization approximation to maximize the computational efficiency during MD simulations. The direct approximation also avoids the possibility of a “polarization catastrophe” inherent to the SCF approach, a numerical instability where two nearby inducible dipoles mutually polarize each other without limit. The current proof-of-concept models are trained for carbon, hydrogen, oxygen, and nitrogen, but our workflows can be easily applied to the full range of elements in drug-like molecules. We demonstrate the utility and accuracy of these models in calculations on isolated molecules and in simulations of organic liquids. We close with a discussion of the next steps to further evaluate these methods and to integrate them into a comprehensive polarizable force field.

## 2 Methods

The overall structure of our direct polarizability model is as follows: each atom has an atom-centered, constant, partial charge as well as an atom-centered, linear, isotropic point-polarizability. In the direct approximation, the point-polarizabilities are felt only by the field generated by the partial charges. That is, they do not feel the other induced dipoles. In accordance with the scaling scheme for short-range intramolecular interactions used in Applequist-like models,^23,24^ we apply a scaling factor *f*_*jk*_ to exclude 1–2 and 1–3 charge–polarizability interactions (*f*_*jk*_ = 0.0), and we scale 1−4 interactions by *f*_*jk*_ = 0.5. These scalings are applied during fitting and also during simulations. Note that 1–2 and 1–3 interatomic distances vary little across MD snapshots, due to the high spring constants of typical bond-stretch and angle-bend terms, so omitting 1–2 and 1–3 charge–polarizability interactions omits a nearly constant term, which may be at least partly adjusted for by suitably chosen partial charges. It is also worth noting that a scaling factor is not needed to avoid polarization catastrophe because the direct approximation does not have this numerical instability, so *f*_*jk*_ could be safely treated as an adjustable parameter to further optimize short-range electrostatics.

### 2.1 Optimization of Typed Polarizabilities

Both polarizable electrostatic models developed here (Sections 2.2 and 2.3) use typed polarizabilities, and we consider two typing schemes. A first, minimalist model applies a single value of polarizability to all atoms of a given element (C, H, O, and N). These are termed element-based polarizabilities. The second, more fine-grained, typing scheme applies the same polarizability to all atoms of the same Lennard–Jones (LJ) type, using LJ types from the Open Force Field (OpenFF) Sage force field.^4^ These are termed Sage LJ-based polarizabilities. The types are detected with SMARTS patterns^47^ and parametrized in the SMIRKS Native Open Force Field (SMIRNOFF) format.^48^ Polarizabilities associated with both typing schemes are trained based on a training set of 39 compounds, each in an average of seven conformations (minimum 4 and maximum 11) differing from all others by at least 0.5 Å root-mean-square deviation (RMSD) (Section 2.4), to reduce concerns about conformational dependencies in our ESP fits.^5,49^

To train the polarizabilities, we computed baseline QM ESPs, *V*_QM,*ik*_, for each conformer *k* in the training set, where *i* indexes the *m*_*k*_ ESP sampling points around the conformer. For each conformer, we also computed six polarized QM ESPs by imposing uniform external fields of magnitude 0.01 au in the +*x*, −*x*, +*y*, −*y*, +*z*, −*z* directions and computed their differences relative to the baseline QM ESPs, *V*_diff,*ikl*_, where *l* ∈ [1, ..., 6] indexes the field directions. Using different field directions allows for averaging over any anisotropy in the induced polarization. The field strength of 0.01 au was chosen to approximate the external electric field generated by a sodium ion about 4 Å away. We used global optimization with the Nelder−Mead method in SciPy^50^ to find typed polarizabilities that minimize the following error function
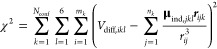
2where *N*_conf_ is the number of conformations of all molecules in the training set, *n*_*k*_ is the number of atoms in the molecule corresponding to conformation *k*, **μ**_ind,*jkl*_ is the induced dipole on atom *j* of conformer *k* with external field direction *l*, and ***r***_*ijk*_ is the vector from atom *j* to ESP point *i* for conformer *k*. The second quantity in parentheses is the potential at grid point *i* generated by the induced dipoles. Because we are using the direct approximation and are fitting polarizabilities to differences in ESP generated by an inducing field, the induced dipoles in eq 2 are given simply by
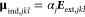
3where α_*j*_ is the typed polarizability assigned to atom *j,* and ***E***_ext,*jkl*_ is the external field at atom *j* for field direction *l*. Because we are using typed polarizabilities, this polarizability is the same for all instances of the polarizability atom type across all conformers (*N*_conf_) of all molecules.

The typed polarizabilities provided by this procedure are independent of the choice of atomic partial charges and therefore allow users to choose among charge-assignment methods: here RESP-dPol and AM1-BCC-dPol. Unlike fitting to molecular dipole moments or molecular polarizabilities, deriving polarizabilities from QM ESPs follows what has arguably been the most successful approach for deriving atomic partial charges (RESP, AM1-BCC). This approach makes physical sense as it emphasizes the influence of polarization on the strong, short-range intermolecular interactions that are particularly relevant for condensed phase simulations.

### 2.2 RESP-dPol Charge Model

The RESP-dPol model assigns partial charges with a RESP-like fitting method in the context of the type polarizabilities discussed above. It differs from the standard RESP because the induced dipoles also contribute to the computed ESPs. Pioneering work by Cieplak et al.^43^ used a similar methodology and achieved reasonable agreement with experimental solvation free energies. In RESP-dPol, we adopt the two-stage hyperbolic restraints used in RESP to reduce nonphysical variations of partial charges.^5^ The first stage is used to make sure all polar regions are well-fitted, and a weak restraint (*a* = 0.005 au, *b* = 0.1 au) is used to decrease the overall magnitude of all partial charges. In the second stage, all partial charges in polar regions are fixed, and forced symmetry restraints and a strong hyperbolic restraint (*a* = 0.01 au, *b* = 0.1 au) are used to achieve an optimal description of electrostatics.

We created a toolkit (Section 2.5) that derives RESP-dPol charges for a given molecule in a given conformer by fitting to its baseline QM ESP (*V*_QM,*i*_), i.e., its ESP in the absence of an external field, by minimizing the following quantity with a least-squares procedure
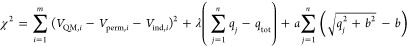
4where *n* is the number of atoms,  is the Coulomb potential at grid point *i* generated by the partial charges, and *V*_ind,*i*_ is the contribution from induced dipoles in the direct approximation
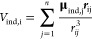
5a
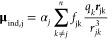
5b

A Lagrange multiplier, λ, is used to constrain the sum of atomic charges (*q*_*j*_) to the correct molecular charge, *q*_tot_.^49,51^ The last term in eq 4 is the hyperbolic restraint described above.

### 2.3 AM1-BCC-dPol Charge Model

The AM1-BCC-dPol model generates partial charges similar in quality to those of RESP-dPol, but without the burden of computing QM ESPs and fitting partial charges to them for each new molecule. Just as in the standard AM1-BCC method,^6,7^ AM1-BCC-dPol charges are constructed as a sum of population precharges (*q*_*j*_^pre^) generated with the fast AM1 method^52^ and bond charge correction (BCC) parameters (***B***_β_) predefined based on bond connectivity analysis (***T***_*j*β_)
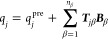
6where the summation of the BCC-dPol correction term runs over the total number of bond types (*n*_β_). We used the same BCC types as used in the standard AM1-BCC method.

The training of BCC-dPol parameters is similar to the derivation of RESP-dPol charges. Both permanent charges and induced dipoles contribute to ESPs on grid points, and the BCCs are adjusted to minimize deviations from baseline QM ESPs. The contribution to χ^2^ from one conformer of one molecule is given by
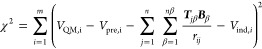
7where *V*_pre,*i*_ is the contribution to the computed ESP at site *i* calculated with AM1 population charges, and *V*_ind,*i*_ is the contribution from induced dipoles. The BCCs are fitted by minimizing the sum of χ^2^ over all conformers of a training set of 119 molecules (Section 2.4), much as detailed in eq 2. Once the BCCs have been optimized, the AM1-BCC-dPol method allows both polarizabilities and polarization-consistent partial charges to be assigned to a new molecule as quickly and easily as partial charges alone are assigned with the traditional AM1-BCC method.

### 2.4 Training Sets and Reference QM Data

The polarizability training set consists of 39 small molecules from the RESP2^53^ training data set. An additional set of 80 small molecules from the OpenFF BCC refit study (COH data set),^54^ for a total of 119 molecules, were added to train BCC-dPol parameters (Figure S4).

All molecules used consist only of elements C, H, O, and N, but a diverse range of chemical fragments, including aliphatic carboxylic acids, amides, aldehydes, and aromatic hydrocarbons, are included to ensure the transferability of the resulting parameters. Two charged molecules are included in the training sets to represent electrostatic environments caused by charged proteins or ligands. For molecules with rotatable bonds, multiple conformations are generated and fitted as if they were independent molecules. Baseline and polarized QM ESPs were evaluated at the MP2/aug-cc-pVTZ^55,56^ QM level of theory, because prior benchmarks of electronic structure methods for molecular mechanics^57,58^ and polarizable force fields^25,30,42^ suggest that this QM method affords a favorable balance of efficiency and accuracy. The external electric fields used to generate polarized ESPs have a strength of 0.01 au ESPs were computed at Merz–Singh–Kollman (MSK) style grid points,^51,59^ located outside the van der Waals radii of all atoms using a spacing of 0.126 Å and a density of 17 points per Å^2^ for a total of 10 layers.

### 2.5 Infrastructure for Parametrization

The optimization of polarizabilities and charge models was implemented in an open-source Python package, Fast atom-centered typed isotropic ready-to-use polarizable electrostatic model (Factor-Pol).^60^

**Figure 1:**
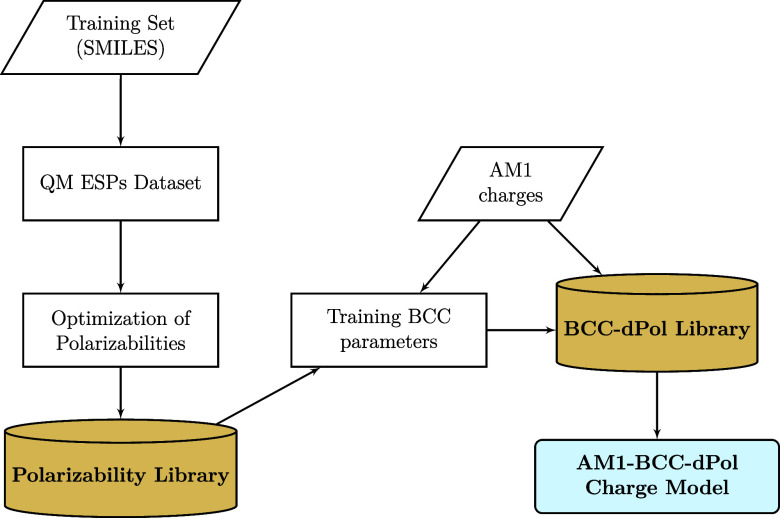
Diagram of the Factor-Pol toolkit used to carry out QM calculations, derive optimized, typed polarizabilities and polarization-adapted BCCs, and assign RESP-dPol and AM1-BCC-dPol charges, and polarizabilities to molecules.

As illustrated in Figure 1, Factor-Pol combines QM calculations, optimization, and parametrization for the current and continued development of the present polarizable electrostatic models. The Factor-Pol package also includes an interface to the OpenFF software infrastructure for the future development of a full polarizable force field, including tuned LJ parameters, torsion parameters, and a polarizable water model. Factor-Pol is also used to assign RESP-dPol and AM1-BCC-dPol charges and typed polarizabilities to molecules for use in MD simulations with OpenMM and the MPID (multipole and induced dipole) plugin.^61,62^

### 2.6 Benchmarking of Electrostatics Models

#### 2.6.1 Accuracy of Baseline Molecular ESPs

We evaluated the accuracy of both RESP-dPol and AM1-BCC-dPol in terms of relative root-mean-square errors (RRMSEs) to baseline gas-phase QM ESPs on a test data set (Figure S3).

As done previously,^5^ RRMSE was computed as eq 8
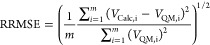
8where *m* is the number of grid points around a molecule, and *i* indexes each grid point.

#### 2.6.2 Gas Phase Molecular Dipole Moments

We also evaluated accuracy by comparing the gas-phase molecular dipole moments they yield to corresponding gas-phase QM dipole moments evaluated at the MP2/aug-cc-pVTZ level of theory on the molecules used to train BCC-dPol parameters (Figure S4). It is generally believed that gas-phase partial charges derived from HF/6-31G* QM level of theory are overpolarized for the gas-phase and are therefore fortuitously suitable for use in the condensed phase.^51^ However, a fixed, overpolarized electrostatic model is not ideal for use to simulate properties that involve large changes in the electrostatic environment of a molecule, such as the transfer free energies of a molecule from vacuum to water. The RESP-dPol and AM1-BCC-dPol models include explicit polarizability and thus, like other polarizable models, should be able to account for such changes in the electrostatic environment. We, therefore, would like them to generate accurate, rather than overpolarized, gas-phase molecular dipole moments. The gas-phase permanent molecular dipole moments from our models were calculated as
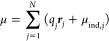
9where *N* is the number of atoms in the molecule, and ***r***_*j*_ and μ_ind,*j*_ are, respectively, the coordinates and induced dipole of atom *j*.

#### 2.6.3 Condensed Phase Molecular Polarizability

The polarizability of a molecule, α_*M*_, can be approximated from the measured refractive index, *n*, of its liquid form via the Lorentz–Lorenz equation^63,64^
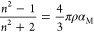
10Here, ρ is the number density of molecules in the liquid. The present polarizability model treats each atom of a molecule as having an isotropic polarizability α_*j*_ that “feels” only permanent charges and any external field that may be present, so the molecular polarizability is simply
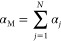
11where *N* is the number of atoms.

Molecular polarizabilities have been used to derive atomic polarizabilities with an additivity method in previous work.^65−67^ Here, instead, we use experimental molecular polarizabilities as an efficient check of the magnitude of our QM-derived atomic polarizabilities. Reference experimental data were obtained from previous compilations.^66,68^

#### 2.6.4 Condensed Phase Properties of Organic Liquids

We used simulations to compute the mass densities and dielectric constants of 17 polar and nonpolar organic liquids by using the AM1-BCC-dPol electrostatic model and compared the results to those obtained from matched simulations by using the fixed-charge AM1-BCC electrostatic model. Condensed-phase simulations of these liquids were performed with OpenMM.^69^ The MPID plugin^61^ was used to enable calculations with AM1-BCC-dPol and typed polarizabilities, using the keyword “direct” to enable direct polarization. For all liquid systems, the valence terms and van der Waals terms were assigned from the OpenFF Sage force field.^4^ Cubic liquid boxes composed of 256 molecules were built with the PACKMOL package,^70^ as simulation systems. Scripts to set up simulations are available online.^71^

Each system was equilibrated with 5 ns simulations in the *NVT* ensemble, followed by 15 ns *NPT* production using a time step of 2 fs. MD trajectories for postprocessing were saved every 1 ps. For a given MD trajectory, the mass density was evaluated as
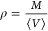
12where *M* is the total mass of the system, *V* is the volume of the simulation box, and the angle brackets indicate the average over simulation snapshots.

The dielectric constant of a condensed system is a key factor in determining the strength of the electrostatic interactions. Simulations with fixed-charge electrostatic models are notorious for underestimating the dielectric constants of nonpolar liquids because this derives almost entirely from electronic polarization. In contrast, the dielectric constants of polar liquids are largely derived from orientational polarizability, which can be captured reasonably well by a force field that lacks an explicit treatment of electronic polarization. Here, we computed the static dielectric constants of liquids of varying polarity using a dipole fluctuation approach^19,20,72^
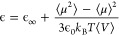
13where μ is the total dipole moment of the system, and ϵ_∞_ is the high-frequency dielectric constant
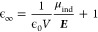
14where ***E*** is the local electric field. Because of the simplicity of the direct polarization approximation, the high-frequency dielectric constant can be obtained analytically as
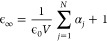
15

## 3 Results

### 3.1 Quality-of-Fit to QM ESPs

The ability to reproduce accurate QM ESPs around a given molecule is essential for reproducing the intermolecular interactions with the surrounding molecules for simulations of complex condensed systems. We examined the ability of two polarizability typing schemes, one based simply on elements and the other assigning a separate polarizability to each LJ atom type in the OpenFF Sage force field. Each typing scheme was tested with two charge models, RESP-dPol and AM1-BCC-dPol, both optimized to reproduce baseline gas-phase QM ESPs of training-set molecules in the presence of our typed polarizabilities.

Since RESP-dPol charges are derived by directly fitting to QM ESPs, they afford optimal accuracy on this test, and the comparison with AM1-BCC-dPol provides information on whether the fast AM1-BCC-dPol charge model is suitable for use in condensed-phase simulations. It is worth mentioning that the RESP-dPol charge model does not use a training set, and we computed RRMS results for RESP-dPol charges on the training set and test set only for comparison with the AM1-BCC-dPol charge model. Therefore, it is possible for RESP-dPol to generate lower RRMS errors on the test set than on the training set.

Table 1 shows that AM1-BCC-dPol yields RRMS errors relative to QM ESPs that are only marginally higher than those provided by RESP-dPol. This favorable result demonstrates that AM1-BCC-dPol partial charges, not fitted to the QM ESPs of each new molecule, are almost as accurate as RESP-dPol charges, which are fitted to each new molecule’s QM ESPs. In addition, the AM1-BCC-dPol errors for the test set are very similar to those for the training set for both element-based polarizabilities and Sage LJ-based polarizabilities. We note, too, that the RRMS errors of RESP-dPol and AM1-BCC-dPol charge models are similar to those reported for standard RESP charges,^5^ indicating that adding polarizabilities does not degrade accuracy. These results suggest that our AM1-BCC-dPol charge model is transferable and suitable to be used as a fast, high-quality alternative to RESP-dPol in condensed-phase MD simulations.

**Table 1:** Quality-Of-Fit of RESP-dPol and AM1-BCC-dPol Electrostatic Models to Baseline QM ESPs

polarizability types	charge model	RRMS (%)	
		training set	test set
element	RESP-dPol	0.13	0.12
element	AM1-BCC-dPol	0.18	0.22
Sage LJ	RESP-dPol	0.13	0.13
Sage LJ	AM1-BCC-dPol	0.21	0.21

The Sage LJ-based polarizabilities are, in principle, tuned for the chemical environment of each atom in a molecule, whereas the element-based polarizabilities do not depend on chemical environments. Thus, Sage LJ types might be expected to provide a more accurate description of electronic polarization. Nonetheless, the RRMS errors relative to QM ESPs from the two typing schemes are essentially indistinguishable. In particular, the element-based and Sage LJ-based electrostatic models perform almost identically on the test set, although the element-based set has only four polarizabilities, whereas the Sage LJ-based set has 14 polarizabilities. We conclude that the minimalist elemental-based polarizability types, paired with the AM1-BCC-dPol charge model, may be sufficient for an accurate polarizable electrostatic model.

### 3.2 Molecular Dipole Moments

Previous benchmarking studies on electronic structure methods for gas-phase dipole moments^57,58^ suggest that HF/6-31G* overpolarizes molecules, and MP2/aug-cc-pVTZ predicts accurate molecular dipole moments. Although the overpolarization that results from fitting partial charges to QM ESPs at the HF/6-31G* level is generally considered a fortuitous outcome in the RESP and AM1-BCC charge models, as it yields charges suitable for use in condensed-phase simulations, it is not ideal for simulations at low dielectric environments, such as in the gas phase or inside protein cavities. For the present polarizable model, where polarity adapts to the environment, we hope to see gas-phase molecular dipole moments that accurately match accurate gas-phase QM dipole moments provided by the reliable MP2/aug-cc-pVTZ level of theory.

**Figure 2:**
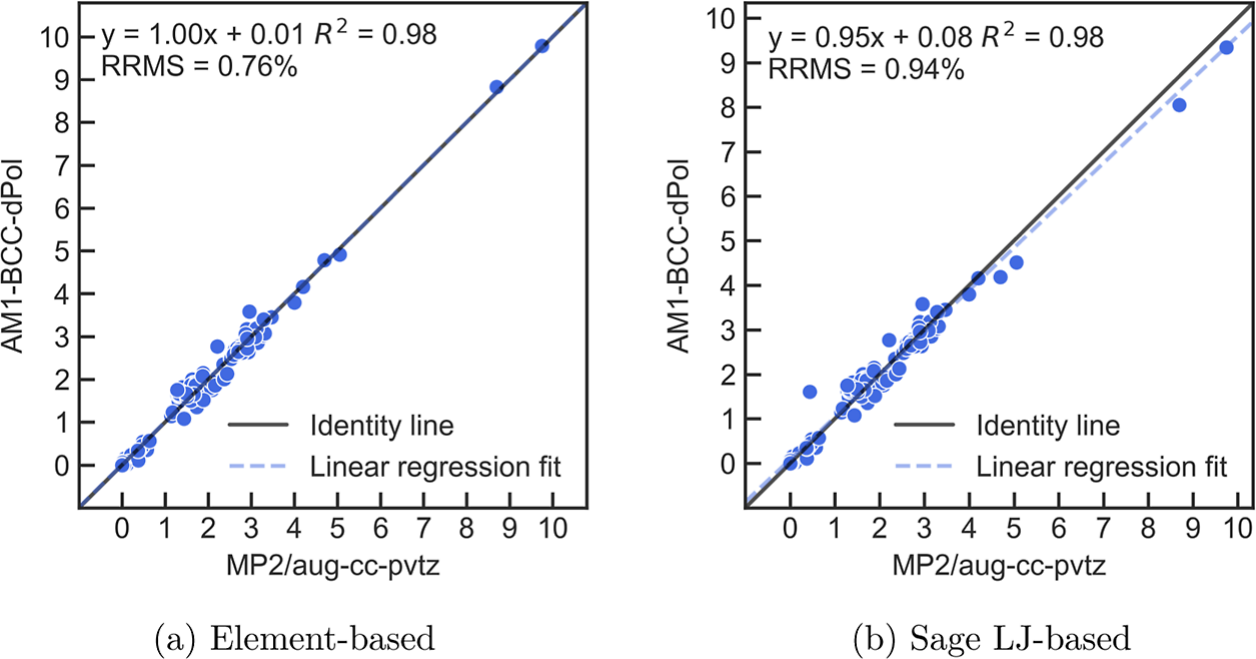
Comparison of gas-phase molecular dipole moments (Debye) computed with the AM1-BCC-dPol electrostatics model with QM reference results for element-based polarizabilities (a) and Sage LJ type-based polarizabilities (b).

As shown in Figure 2, molecular dipole moments calculated for 107 neutral molecules with the AM1-BCC-dPol model, using both the element-type and Sage LJ-type polarizabilities, agree extremely well with the reference QM results, with relative errors below 1%. Interestingly, the element-based typing scheme gives a slightly lower RRMS error and a linear regression slope slightly closer to unity. Thus, both variants of the AM1-BCC-dPol electrostatics model accurately reproduce reference gas-phase dipole moments, as hoped.

### 3.3 Condensed Phase Molecular Polarizabilities

The present atomic polarizabilities were derived to best fit QM ESPs and thus capture the intermolecular interactions of a system in a dynamic electrostatic environment. However, there is no guarantee that these polarizability will also yield accurate molecular polarizabilities. Comparing with these experimental observables thus offers an independent check of the robustness and transferability of our model.

**Figure 3:**
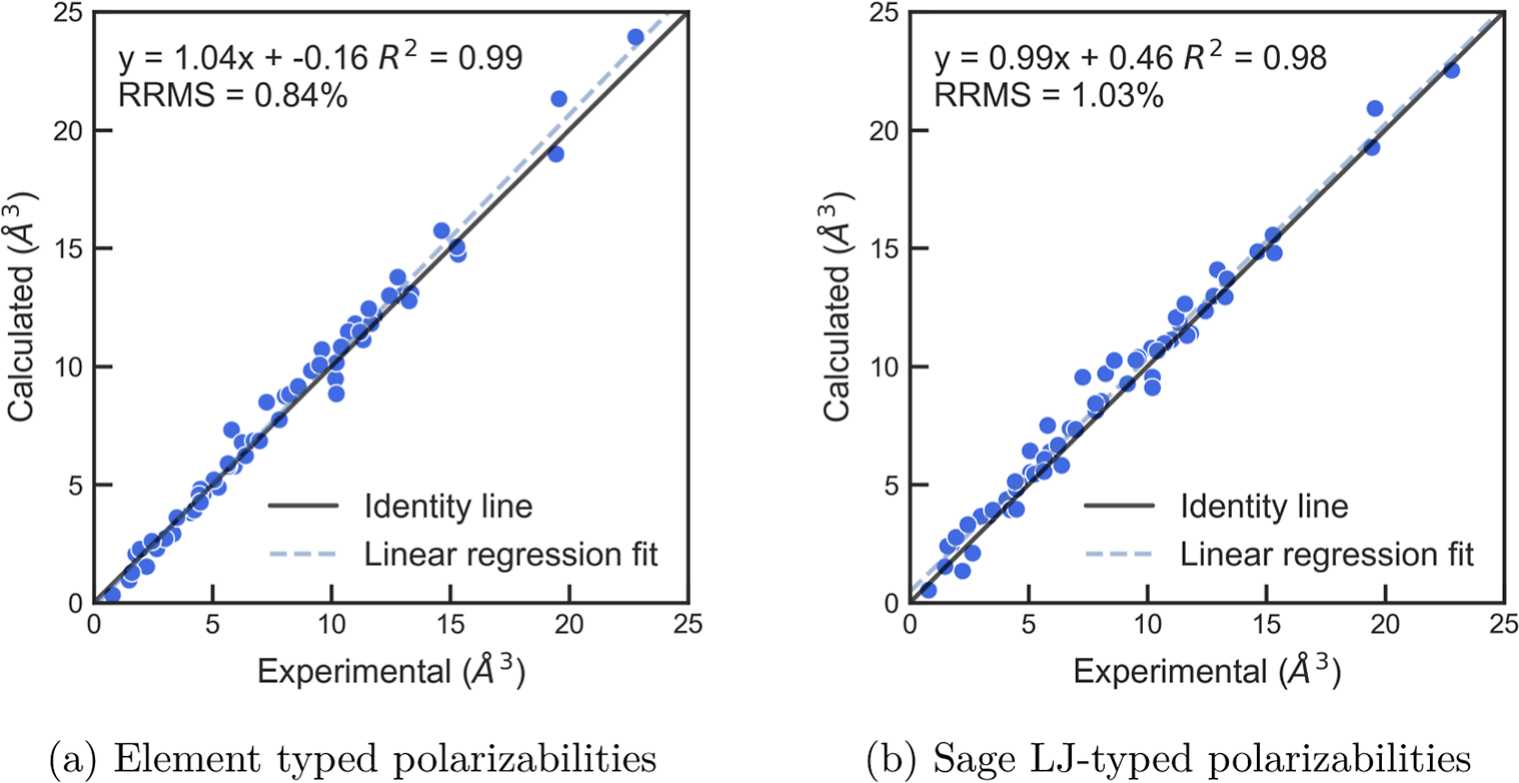
Comparison of molecular polarizabilities computed from our atomic polarizabilities against experimental reference data, for element-based polarizabilities (a) and Sage LJ type-based polarizabilities (b).

We find that molecular polarizabilities computed from both element-based and Sage LJ-type atomic polarizabilities (eq 11) agree well with those derived from experimental indices of refraction, as shown in Figure 3. It is worth noting that, although the element-typed polarizabilities include four types, the minimalist element-typed polarizabilities transfer well to molecules with diverse chemical environments that are not included in the training data, such as nitriles and alkynes. The chemical structures of molecules with available experimental measurements are presented in Figure S5. These results suggest that our atomic polarizabilities based on induced gas-phase QM ESPs retain their broader physical meaning and can be valid for use in molecular simulations.

### 3.4 Transferability of AM1-BCC-dPol across Molecular Conformations

**Figure 4:**
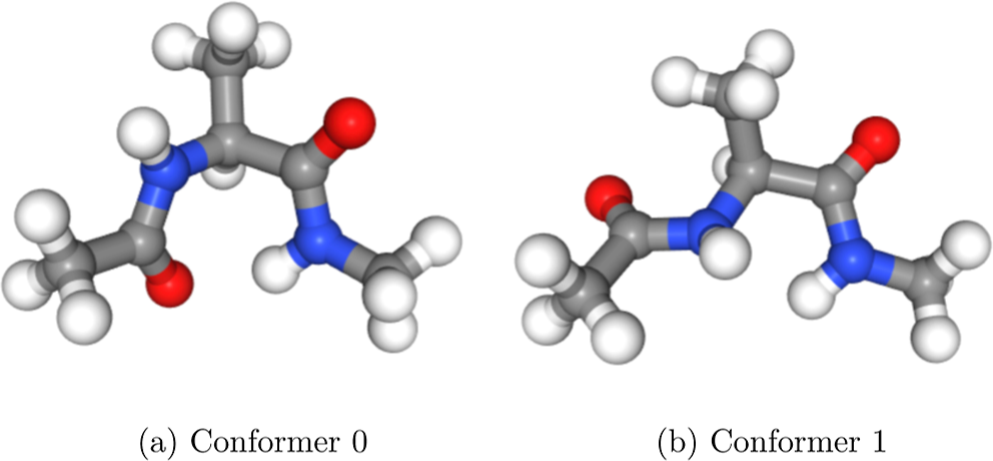
Two conformers of alanine dipeptide used to examine the transferability of charges trained for one conformation and tested on a second conformation (see Table 2).

For a fixed charge model, the partial charges that best replicate QM ESPs for one conformation of a molecule are not optimal for other conformations, and this problem is usually referred to as conformational dependency.^5,49^ Thus, losses in accuracy are inevitable when fixed charge models are used in simulations in which the molecular conformation varies. We conjectured that the polarizable AM1-BCC-dPol and RESP-dPol model would better preserve accuracy across multiple conformations of a molecule compared to the nonpolarizable AM1-BCC model because of the ability of the inducible dipoles to respond to changes in the field due to changes in conformation. As an illustrative test of this idea, we examined the transferability of the AM1-BCC-dPol and RESP-dPol electrostatic models across two conformations of alanine dipeptide, conformer 0 with an intramolecular hydrogen bond (Figure 4a), and conformer 1 without (Figure 4b) an intramolecular hydrogen bond. Table 2 shows the average unitless RRMS errors of gas-phase ESPs computed with conformer 0 and evaluated on conformers 0 and 1, and vice versa, for the nonpolarizable AM1-BCC model, and the polarizable models AM1-BCC-dPol and RESP-dPol.

**Table 2:** Average RRMS Errors (Unitless) of ESPs, Relative to Reference QM ESPs, Computed Using Partial Charges Derived from One Conformer (Column 1) and Tested for a Second Conformer (Columns 2 and 3), for Electrostatic Models AM1-BCC, AM1-BCC-dPol, and RESP.

AM1-BCC-dPol	conformer 0	conformer 1
conformer 0	0.82	0.97
conformer 1	0.80	0.75

RESP-dPol	conformer 0	conformer 1
conformer 0	0.63	1.19
conformer 1	0.93	0.75

AM1-BCC	conformer 0	conformer 1
conformer 0	0.95	1.28
conformer 1	0.87	1.13

As shown in Table 2, AM1-BCC-dPol provides consistently low errors (<1%) across both the training and test conformers. RESP-dPol provides slightly greater accuracy overall when trained and tested on the same conformation (diagonal elements of each subtable), but less accuracy when trained on one conformer and tested against the conformer (off-diagonal elements). These increased errors presumably reflect overtraining. The nonpolarizable AM1-BCC model is less accurate than AM1-BCC-dPol in all four cases and less accurate than RESP-dPol in all but one case. Overall, these results suggest that our AM1-BCC-dPol model is more transferable and accurate than AM1-BCC, at least in part because its inducible dipoles respond appropriately to changing electrostatic environments. The simplicity, transferability, and accuracy afforded by AM1-BCC-dPol make it an attractive charge model for use in MD simulations where conformational charges play an important role.

### 3.5 Properties of Organic Liquids from Simulations

We ran simulations of 17 organic liquids (Figure S1) at constant pressure to compare the ability of force fields using various electrostatics models to provide accurate mass densities and dielectric constants. We tested the fixed charge AM1-BCC model, AM1-BCC-dPol with element-based polarizability types, and AM1-BCC-dPol with Sage LJ-based polarizability types. We drew all other force field parameters (Lennard–Jones and valence terms) from the OpenFF, Sage force field. As previously done,^73^ we focused on the reciprocals (*D*^−1^) of the dielectric constant (*D*). This approach is motivated by the fact that electrostatic energies are proportional not to the dielectric constant but to its reciprocal
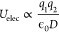
16where ϵ_0_ is the permittivity of free space and *D* is the relative dielectric constant.

**Figure 5:**
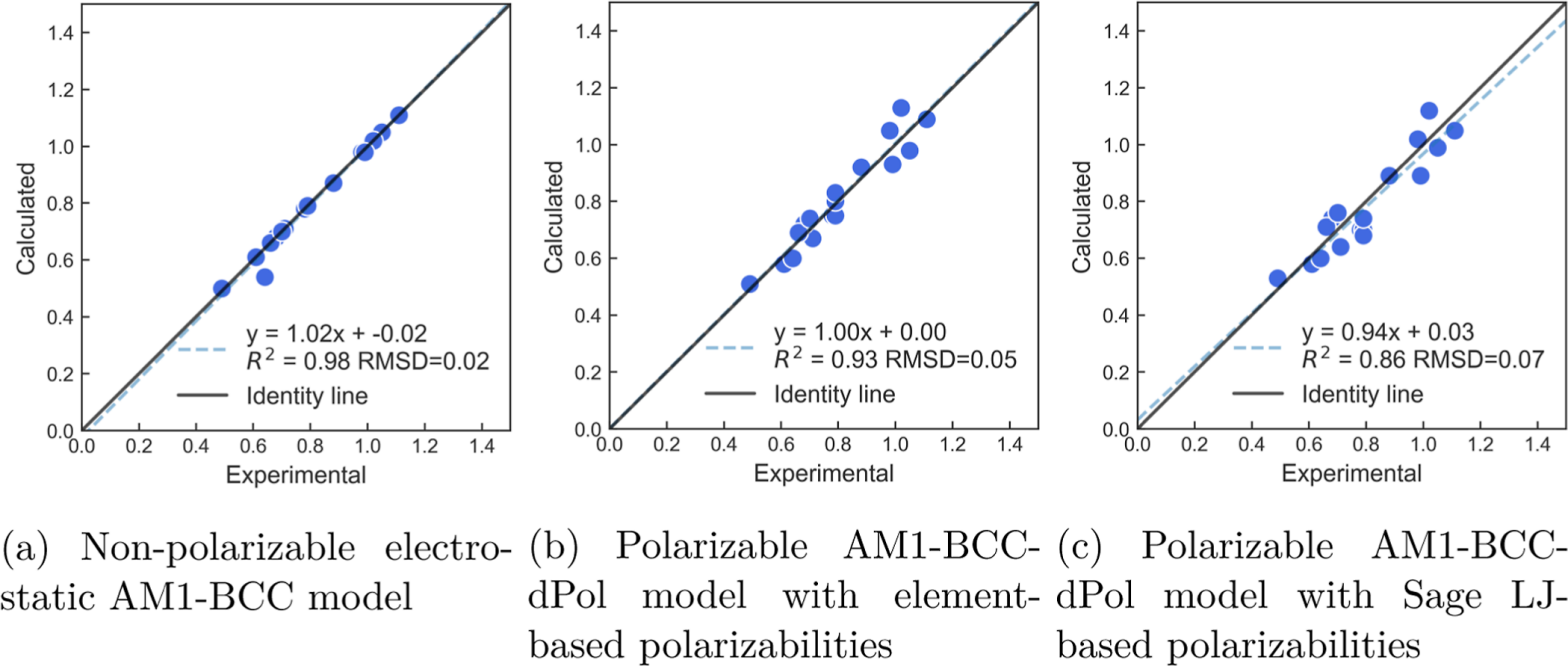
Comparison of computed and experimental mass densities (g/mL) for simulations with the AM1-BCC fixed-charge model (a) and with our AM1-BCC-dPol electrostatic model using element-based polarizabilities (b) and Sage LJ-based polarizabilities (c).

All three electrostatics models (AM1-BCC, AM1-BCC-dPol with element-based polarizability types, and AM1-BCC-dPol with Sage LJ types) give good agreement with experimental mass densities (Figure 5 and Table 3) with RMS errors of 0.03, 0.05, and 0.07 g/mL, respectively. Compared to results from the nonpolarizable Sage force field, liquid densities computed with polarizable electrostatic models are somewhat less accurate, but this is as expected given that the Sage FF was fitted against liquid state data using the nonpolarizable AM1-BCC electrostatics model. We anticipate that retraining the LJ and torsional terms against liquid state data in the context of AM1-BCC-dPol will lead to liquid densities as accurate as those obtained with the nonpolarizable AM1-BCC model.

**Table 3:** Condensed Phase Mass Densities (g/mL) and Inverse Dielectric Constants (1/*D*) of Organic Liquids from Simulations and Experiments Computed with the Following Electrostatics Models: Element-Type AM1-BCC-dPol (E-dPol), Sage LJ-Type AM1-BCC-dPol (S-dPol), and AM1-BCC (noPol)

		density				inverse dielectric constant			
liquid	temp (K)	E-dPol	S-dPol	noPol	expt	E-dPol	S-dPol	noPol	expt
acetic acid	293.15	0.98	0.99	1.05	1.05	0.18	0.19	0.21	0.16
propan-2-one	298.15	0.75	0.70	0.78	0.78	0.08	0.19	0.21	0.05
ether	293.15	0.67	0.64	0.71	0.71	0.23	0.47	0.46	0.23
ethane-1,2-diol	293.15	1.09	1.05	1.11	1.11	0.05	0.08	0.07	0.03
heptane	293.15	0.72	0.74	0.68	0.68	0.55	0.55	0.99	0.52
hexane	293.15	0.69	0.71	0.66	0.66	0.56	0.56	0.99	0.53
octane	293.15	0.74	0.76	0.70	0.70	0.54	0.54	0.99	0.51
propane	293.15	0.51	0.53	0.50	0.49	0.64	0.64	0.99	0.60
prop-1-ene	220.15	0.58	0.58	0.61	0.61	0.61	0.59	0.98	0.47
pyridine	293.15	1.05	1.02	0.98	0.98	0.04	0.07	0.30	0.08
aniline	293.15	1.13	1.12	1.02	1.02	0.16	0.25	0.35	0.14
methylaniline	300.15	0.93	0.89	0.98	0.99	0.13	0.10	0.36	0.17
methanol	293.15	0.75	0.70	0.79	0.79	0.06	0.10	0.11	0.03
ethanol	293.15	0.80	0.68	0.79	0.79	0.09	0.19	0.14	0.04
2-propanol	293.15	0.83	0.74	0.79	0.79	0.11	0.18	0.19	0.05
benzene	293.15	0.92	0.89	0.87	0.88	0.51	0.52	0.96	0.44
ethane	95.15	0.60	0.60	0.54	0.64	0.59	0.61	0.99	0.52
**RMSD vs Expt**		**0.02**	**0.05**	**0.07**		**0.05**	**0.06**	**0.33**	

**Figure 6:**
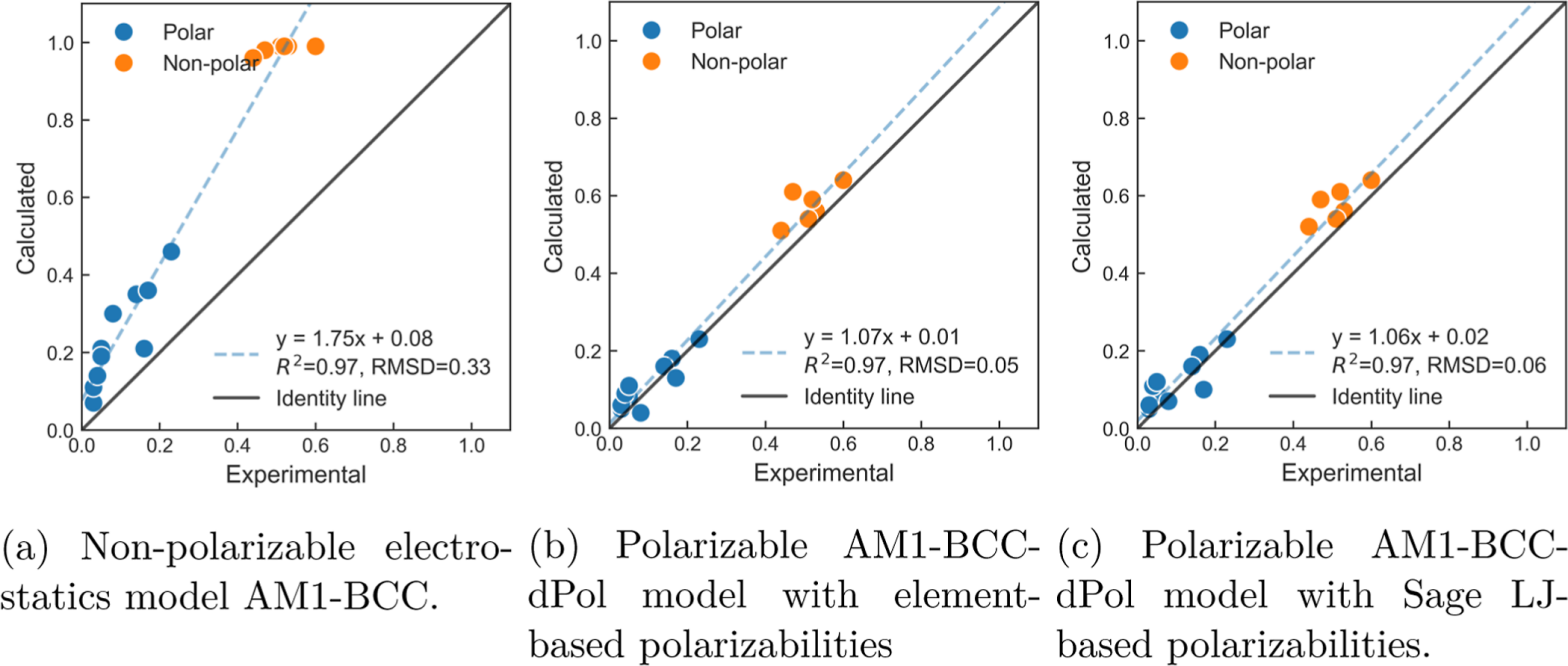
Comparisons of computed and experimental inverse relative dielectric constants (1/*D*), for simulations with the AM1-BCC fixed-charge model (a) and with our AM1-BCC-dPol electrostatic model using element-based polarizabilities (b) and Sage LJ-based polarizabilities (c).

We also find that going from the nonpolarizable AM1-BCC model to the AM1-BCC-dPol models dramatically improves the accuracy of the dielectric constants computed for organic liquids (Figure 6 and Table 3), with the RMSD of the inverse dielectric constant (1/*D*) improving from 0.33 to 0.05 and the slope going from 1.75 to 1.07, in the case of element-based polarizabilities, and similar results for Sage LJ-based polarizabilities. Although improvements are seen across the range of dielectric constants, nonpolar liquids show the greatest improvement. Precisely because they are nonpolar, the orientational polarizability of a nonpolar liquid is negligible, so omitting electronic polarizability causes them to have dielectric constants of about one; i.e., near-zero polarizability. This has the problematic consequence that electrostatic interactions in a nonpolar liquid modeled with a nonpolarizable force field will be overestimated about 2-fold. Including electronic polarizability via the AM1-BCC-dPol model makes these liquids polarizable and corrects the dielectric constants to ∼2. These results suggest that AM1-BCC-dPol will more faithfully model electrostatic interactions in nonpolar media and the lipid membranes of cells.

Interestingly, the dielectric constants of a small range of alcohols are significantly underestimated in the AM1-BCC-dPol simulations (Table 3). Although this underestimation might be traced to inadequate electronic polarizability, this seems unlikely because AM1-BCC-dPol gives good agreement with experimental molecular polarizabilities for these compounds (Table S4). In addition, the dielectric constants of these highly polar liquids are dominated by orientational polarization, so that adding electronic polarization does not have a dramatic effect. This is apparent by comparing AM1-BCC-dPol with the nonpolarizable but otherwise similar Sage force field (Table 3). Note that previous simulations with the nonpolarizable GAFF/AM1-BCC FF similarly underestimate the dielectric constants of these liquids (methanol 20.0, ethanol 11.4, 2-propanol 10.5).^73^ Although all of these underestimates might trace to too-weak partial charges, they may also result from an overly low Kirkwood *g*-factor.^20^ The *g*-factor is the ratio of the liquid’s dipole moment fluctuations to those which would be obtained if there were no correlations among the molecules in the liquid. Thus, a higher *g*-factor gives a higher dielectric constant, other things being equal. Importantly, the *g*-factor depends on not only the electrostatic model but also other force field parameters, such as Lennard-Jones parameters. Thus, correcting these dielectric constants may require a more holistic refitting of the force field.

We chose the direct approximation of polarization primarily for the sake of computational speed relative to a full SCF treatment of inducible dipoles. We compared the average speed of 15 ns *NPT* production simulations using a standard fixed-charge AM1-BCC model, our direct approximation AM1-BCC-dPol, and a full self-consistent mutual polarization model using the same parameters as AM1-BCC-dPol and also executed with the MPID plugin in OpenMM, using keywords “direct” and “mutual” for direct and mutual polarization, respectively. All calculations were run on the same hardware. Simulations with direct polarization average 3.6-fold slower than fixed charge simulations, while simulations with the full mutual polarization calculations, with convergence set to 10^−5^, average 4.5-fold slower. We also compared the speed of the direct approximation with the available OpenMM implementation of the empirical extrapolation scheme for efficient treatment of induced dipoles, namely the OPTn methods,^27,74^ for two sample systems, ethane and ethanol. As detailed in Table S5, the direct method is about twice as fast as the OPT3 and 50% faster than OPT1. (Runs with OPT0, which is theoretically equivalent to the direct polarization, failed for currently unknown reasons and hence are not included here.)

Note that the MPID plugin used here carries along a full anisotropic tensor representation of polarizability and a set of permanent multiples. Thus, we implement our simpler model zeroing the off-diagonal tensor elements and the permanent multipoles, but carrying along these more detailed terms necessarily slows the calculations. We are currently exploring how much further speedup can be attained with a more streamlined implementation. It is worth noting that the OpenMM implementation of the iAMOEBA model achieved a speedup of simulations by a factor of 1.5 to 6 over full mutual polarization in the AMOEBA model, depending on the choices of convergence parameter in the SCF iterations.^29^ Therefore, we expect that greater speed of our direct polarization model will be achievable by further software engineering of the OpenMM polarizability plugin.

## 4 Discussion

We have presented a novel polarizable electrostatic model that utilizes the direct polarization approximation, typed atomic polarizabilities, and a fast AM1-BCC-dPol charge assignment method. A RESP-inspired model, called RESP-dPol, is also available. Our overall approach is designed to capture the key consequences of electronic polarizability in simulations while remaining convenient to use and computationally tractable. The results presented here indicate that the polarizable AM1-BCC-dPol model provides a realistic representation of electrostatics at a moderate computational cost. Its success may be traced, in part, to the fact that, like some of the most widely used fixed-charge electrostatic models, our model is tuned to replicate gas-phase QM ESPs. Calculations of liquid dielectric constants indicate the use of a polarizable electrostatic model is crucial to replicate these important experimental observables, for both nonpolar and polar liquids.

We anticipate that the present approach will enhance the accuracy of simulations for systems where molecules move between regions with very different dielectric constants, such as water and a lipid membrane, or with very different ambient electric fields, such as from water to a protein pocket with many ionized side-chains. These are settings in which large changes in the electronic polarization occur, making it important to account for them in detail. While the current polarizable electrostatic model can be used with valence and van der Waals terms from the existing OpenFF Sage force field in MD simulations, the accuracy of such simulations will be improved by adjusting the LJ and torsional parameters against reference experimental and QM data in the context of the polarizable force field. In the meantime, a compatible and fast polarizable water model is essential for achieving accurate simulations of molecular recognition. The Factor-Pol software infrastructure, that implements both the training and use of these models, is shared as open source at the GitHub repository Factor-Pol.^60^ It can be easily used in the OpenFF software ecosystem, i.e., with OpenFF Toolkit,^75^ OpenFF Evaluator,^76^ and OpenFF Interchange^77^ to enable seamless use with the valence and Lennard-Jones parameters of the OpenFF Sage force field in OpenMM. We have also implemented a version of the Smirnoff-plugin,^78,79^ named as MPID-plugin,^80^ to introduce electronic polarization to the OpenFF force field fitting software stack. This integration sets the stage for planned future work aimed at generating more comprehensive force fields that fully integrate the polarizable electrostatics models. Currently, we are developing a polarizable water model that utilizes direct polarization, atom-centered polarizabilities, and partial charges.

In summary, we have presented a polarizable electrostatic model that is not only fast due to its use of the direct polarization approximation but is both general and convenient to apply due to the use of typed polarizabilities and the AM1-BCC-dPol method for assigning consistent partial charges. We believe that this work provides the foundation for a generally parametrized polarizable force field that can be used in a wide range of applications in biomolecular simulations.

## Supporting Information Available

typed polarizability parameters; MD simulation speed (ns/day) using OpenMM; benchmarking results for molecular polarizabilities; speed comparison of various polarizability models; and chemical structures of molecules used in this work (PDF)
