# Supplementary material for: A Fast, Convenient, Polarizable Electrostatic Model for Molecular Dynamics
Source: J Chem Theory Comput. 2024 Jan 19;20(3):1293–305. doi: 10.1021/acs.jctc.3c01171 (PMC10867846; doi:10.1021/acs.jctc.3c01171)
Supplement: Supplementary file 1 — ct3c01171_si_001.pdf [file ct3c01171_si_001.pdf]

Supporting Information: A fast, convenient, polarizable electrostatic model for molecular dynamics

Liangyue Wang,† Michael Schauperl,‡ David L. Mobley,¶ Christopher Bayly,§ and Michael K. Gilson*,‖

†*Department of Chemistry and Biochemistry, University of California, San Diego, CA 92093, USA*

‡*HotSpot Therapeutics, Inc. Boston, MA 02210, USA*

¶*Department of Pharmaceutical Sciences, University of California, Irvine, CA 92697, USA*

§*OpenEye Scientific, Cadence Molecular Sciences, Santa Fe, NM 87508, USA*

‖*Skaggs School of Pharmacy and Pharmaceutical Sciences, University of California, San Diego, CA 92093, USA*

E-mail: mgilson@ucsd.edu

1 Supporting Information

Contents

1 Supporting Information **S-2**

1.1 Supporting scripts and data S-2

1.2 Polarizability Parameters S-2

1.3 MD Simulation Speed using OpenMM S-4

1.4 Molecular Polarizabilities S-5

1.5 Chemical structure of molecules used in this work S-8

1.1 Supporting scripts and data

Supporting scripts and data to reproduce all data are available at `https://github.com/wwilla7/factorpol-benchmarks-scripts`.

1.2 Polarizability Parameters

Table S1: Element-based polarizabilities (Å^3)

Element Type	Polarizability
C	1.642
H	0.163
O	0.642
N	1.042

Table S2: Sage LJ-typed polarizabilities (Å^3)

SMIRNOFF Pattern	Polarizability
[#1:1]	0.272
[#1:1]-[#6X4]	0.132
[#1:1]-[#6X4]-[A[1]]	0.161
[#1:1]-[#6X4](-[A[1]])-[A[1]]	0.161
[#1:1]-[#6X4](-[A[1]])(-[A[1]]	0.161
[#1:1]-[#6X4]~[*+1,*+2]	0.132
[#1:1]-[#6X3]	0.198
[#1:1]-[#6X3]~[A[1]]	0.271
[#1:1]-[#6X3](~[A[1]])~[A[1]]	0.271
[#1:1]-[#6X2]	0.372
[#1:1]-[#7]	0.040
[#1:1]-[#8]	0.320
[#6:1]	1.579
[#6X2:1]	1.593
[#6X4:1]	1.593
[#8:1]	1.203
[#8X2H0+0:1]	1.301
[#8X2H1+0:1]	1.301
[#7:1]	1.239
[#1]-[#8X2H2+0:1]-[#1]	1.201
[#1:1]-[#8X2H2+0]-[#1]	0.172

[1] A stands for pattern [#7,#8,#9,#16,#17,#35]

1.3 MD Simulation Speed using OpenMM

Table S3: MD simulation speed (ns/day) using OpenMM as MD engine and the MPID plugin for the polarizability models. AM1-BCC-dPol: simulations with AM1-BCC-dpol. AM1-BCC-mPol: matched simulation but with SCF turned on in the MPID plugin. AM1-BCC: simulations with the non-polarizable AM1-BCC model.

SMILE	natoms	AM1-BCC-dPol	AM1-BCC-mPol	AM1-BCC
CO	6	431	351	1287
CC(=O)O	8	404	330	1193
CC	8	384	309	1298
CC=C	9	382	312	1325
CCO	9	381	310	1237
C(CO)O	10	353	297	1210
CC(=O)C	10	371	312	1225
CCC	11	356	288	1228
C1=CC=NC=C1	11	317	262	1229
c1ccccc1	12	387	308	1298
CC(O)C	12	323	260	1310
C1=CC=C(C=C1)N	14	283	239	1339
CCOCC	15	286	239	1233
CNC1=CC=CC=C1	17	288	235	1238
CCCCCC	20	268	224	1151
CCCCCCC	23	231	191	1154
CCCCCCCC	26	224	188	1013
mean	13	334	274	1234

1.4 Molecular Polarizabilities

Table S4: Experimental and calculated molecular polarizabilities (Å^3) using typed atomic polarizabilities.

SMILES	Experimental	Element-based	Sage LJ-based
C(CCO)CO	10.16	9.48	10.78
CC(C)(C)C	10.20	10.17	9.54
CCO	5.08	4.90	5.52
C1=CC=C(C=C1)[N+](=O)[O-]	12.92	12.99	14.11
CCCCCCCCCCCC	22.80	23.94	22.54
C1CCCCC1	11.00	11.81	11.14
N#N	1.76	2.08	2.48
[H][H]	0.79	0.33	0.54
C(=O)N	4.08	3.81	4.37
C1CCCCCCC1	14.63	15.74	14.85
CNC=O	5.91	5.78	6.41
COC	5.24	4.90	5.45
C=C	4.22	3.94	3.95
CCCCCC	11.80	12.13	11.40
CCCCC=C	11.65	11.81	11.31
N#CCC#N	5.79	7.34	7.52
C1C=CC(=O)O1	7.27	8.50	9.55
CCC#N	6.24	6.78	6.68
COC=O	5.05	5.22	6.43
CC=O	4.59	4.58	5.04
CCCC(=O)OC	11.33	11.12	11.86
C1CCCCCC1	12.79	13.78	12.99
CCCCC(=O)OC	13.34	13.09	13.72
CC#N	4.48	4.82	4.82
C	2.65	2.29	2.12
CC(C)(C)C#N	9.59	10.72	10.39

Continued on next page

Table S4 – continued from previous page

SMILES	Experimental	Element-based	Sage LJ-based
CCOCC	10.20	8.84	9.11
CC(=O)N	5.67	5.78	6.09
O	1.49	0.97	1.54
CC(=O)NC	7.82	7.75	8.12
CCC	6.38	6.23	5.83
CC(C)C#N	8.05	8.75	8.53
N	2.22	1.53	1.36
C1CCC=CC1	10.70	11.48	10.98
C1CCCC1	9.15	9.84	9.28
CCCCCCCO	15.33	14.74	14.80
c1ccncc1	9.49	10.07	10.27
CCCCCCO	13.27	12.78	12.95
C1CO1	4.43	4.58	5.13
c1ccc(cc1)O	11.20	11.47	12.08
O=O	1.60	1.28	2.41
[C-]#[O+]	1.95	2.28	2.78
CC	4.48	4.26	3.98
CCCO	6.74	6.87	7.38
CC(C)O	6.97	6.87	7.35
CO	3.32	2.94	3.70
C1CCC(CC1)N	12.44	13.01	12.35
CCCCCCCC(=O)OC	19.44	19.00	19.28
[N-]=[N+]=O	3.00	2.73	3.68
c1ccccc1	10.40	10.83	10.66
C1CC(=O)OC1	8.23	8.83	9.71
C=O	2.45	2.61	3.32
c1ccc(cc1)c2ccccc2	19.57	21.33	20.92
C#C	3.49	3.61	3.93
CCCCCC(=O)OC	15.27	15.06	15.57
C1CC1	5.64	5.90	5.57

Continued on next page

Table S4 – continued from previous page

SMILES	Experimental	Element-based	Sage LJ-based
C1COCCO1	8.60	9.16	10.26
C1CCC(CC1)O	11.56	12.45	12.66
CN(C)C=O	7.81	7.75	8.44

Table S5: Speed comparison of various polarizability models. Calculations were performed with AM1-BCC-dPol and Sage, using the MPID plugin in OpenMM. Units are ns/day.

Systems (256 mols)	OPT1	OPT2	OPT3	Direct Polarization
Ethane	264	228	203	380
Ethanol	273	229	205	376

1.5 Chemical structure of molecules used in this work

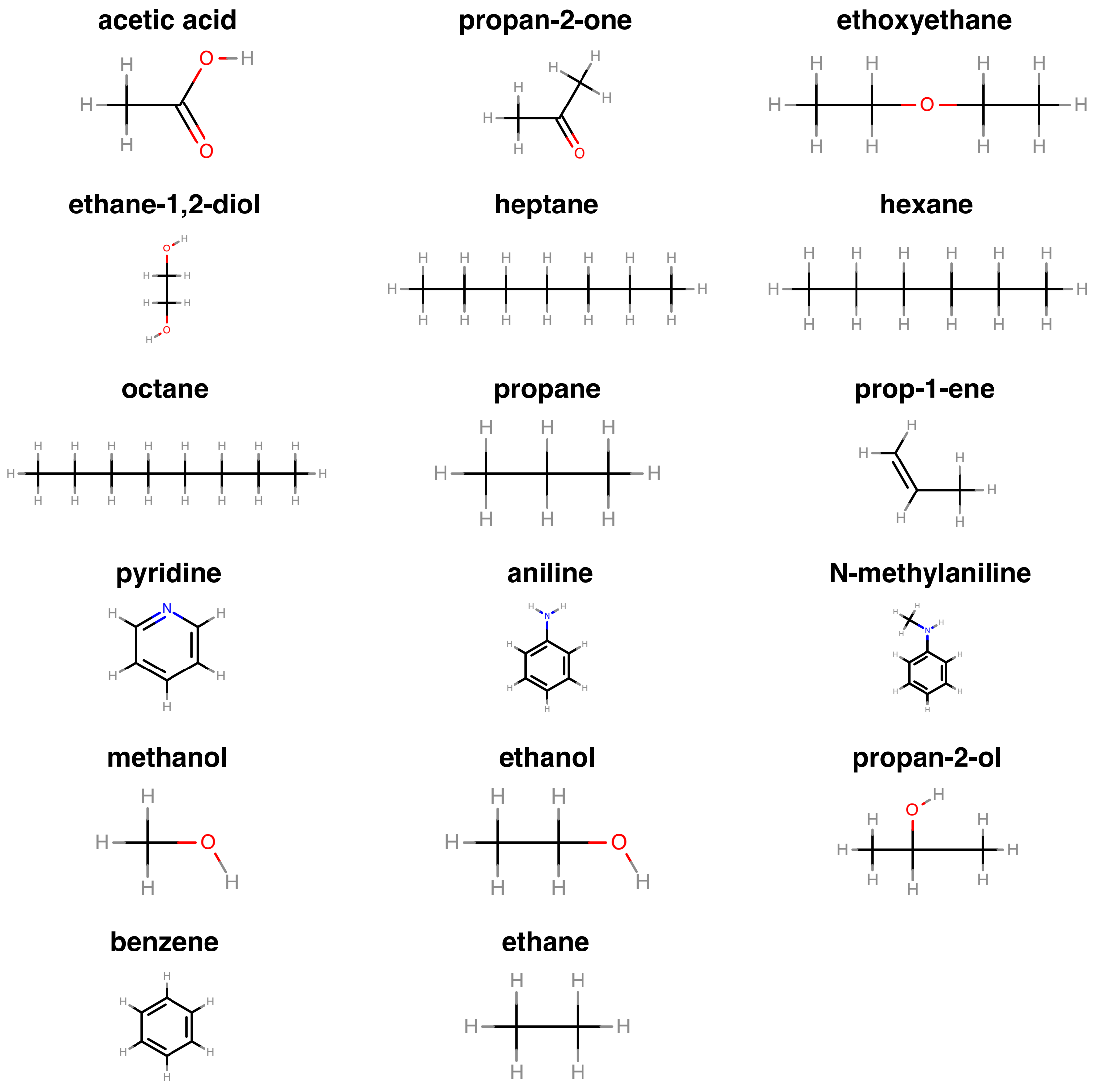

Figure S1: Dielectric constant and density calculation test set

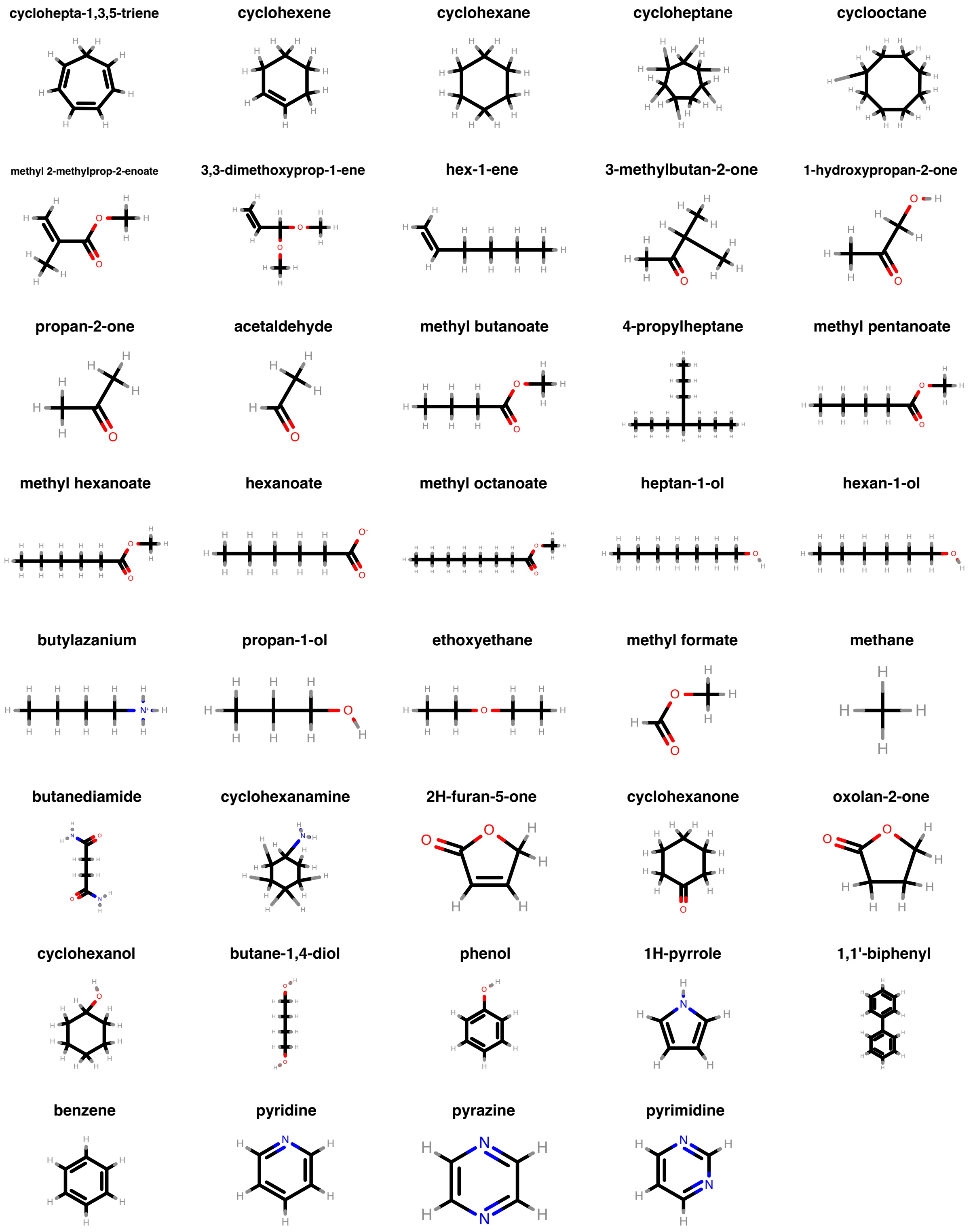

Figure S2: Polarizability training set

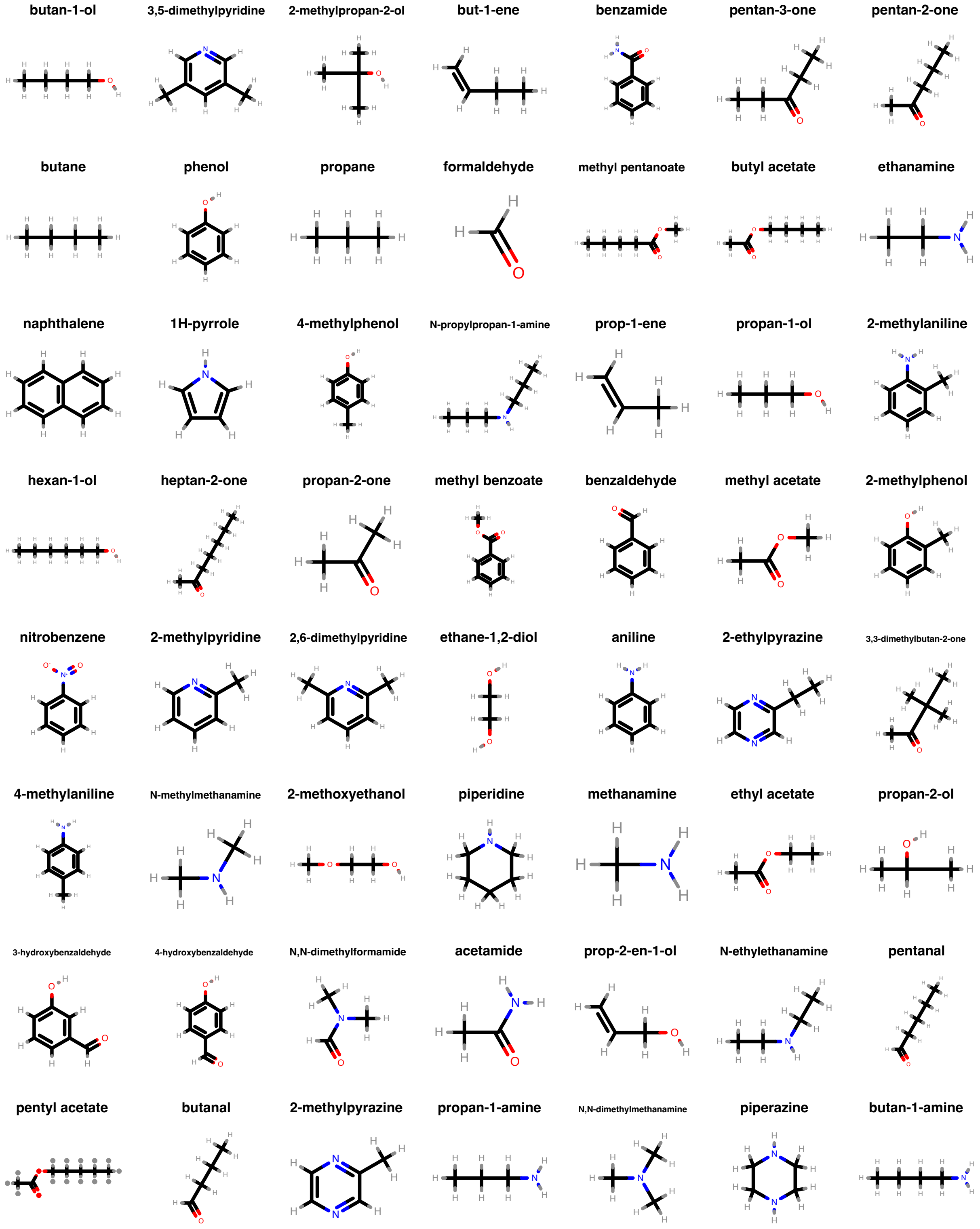

Figure S3: Quality-of-fit to QM ESPs data set

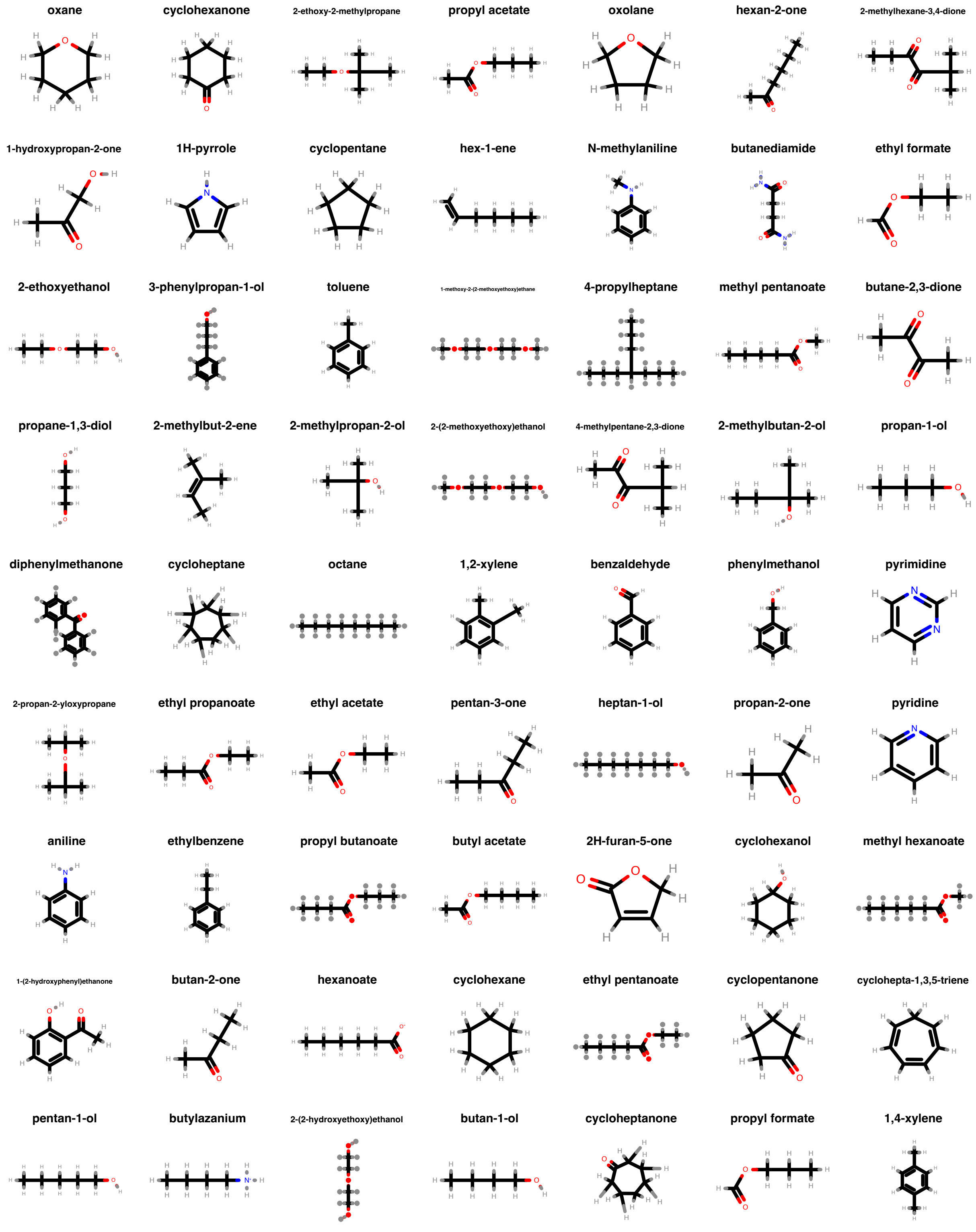

Figure S4: AM1-BCC-dPol library training set

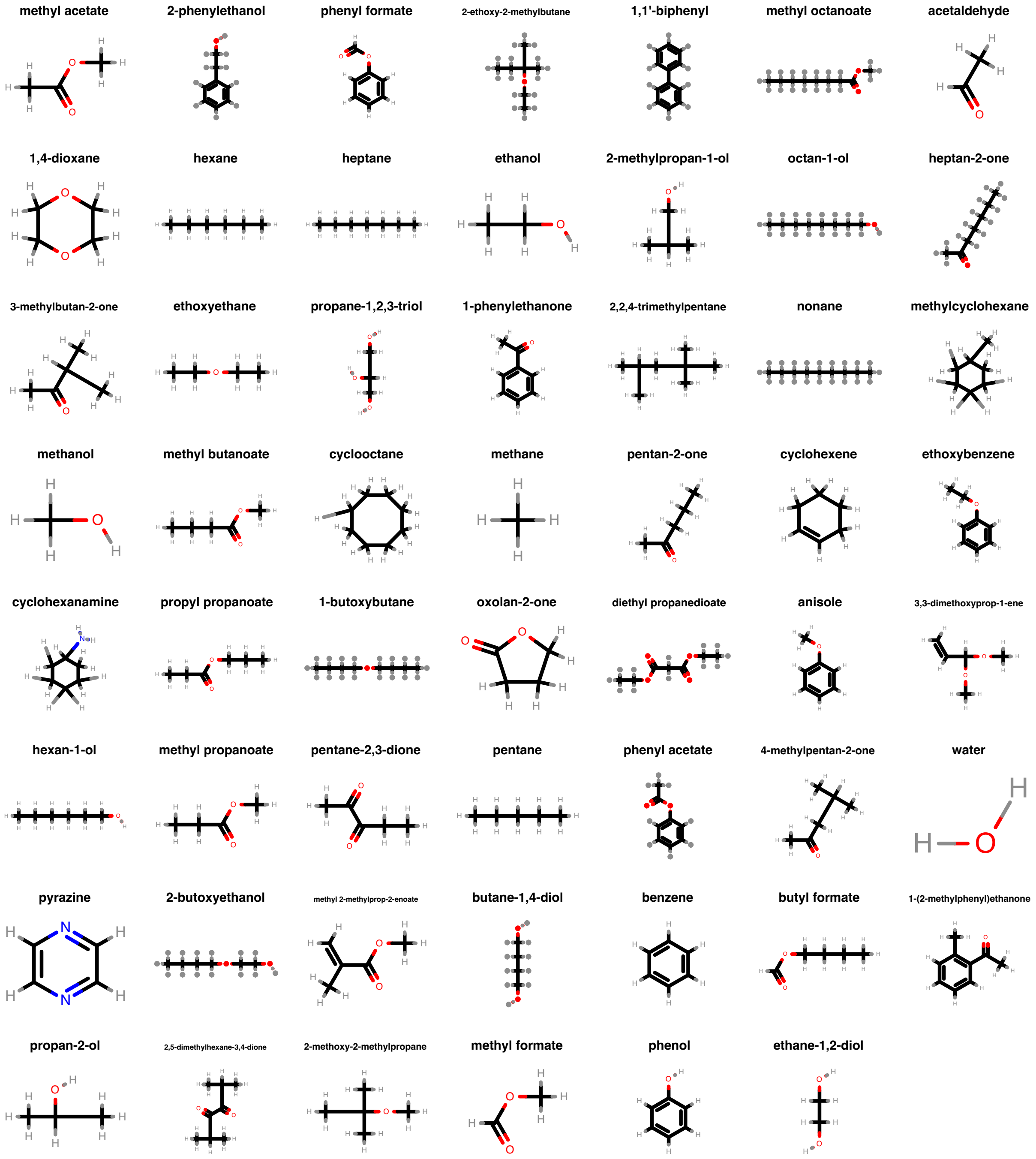

Figure S4: AM1-BCC-dPol library training set (Continued)

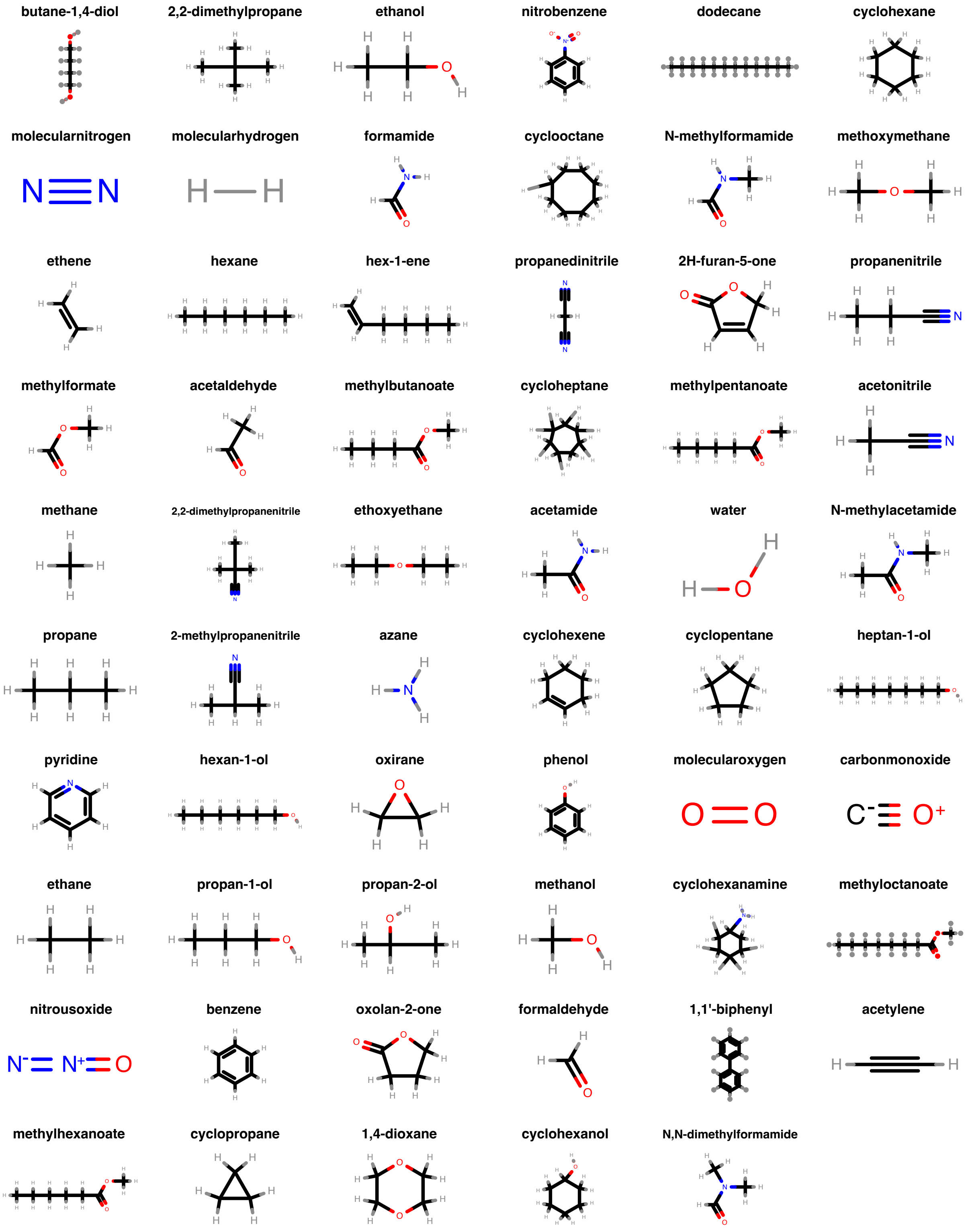

Figure S5: Molecular polarizability data set
